# Effect of Ceramic Surface Treatment and Adhesive Systems on Bond Strength of Metallic Brackets

**DOI:** 10.1155/2020/7286528

**Published:** 2020-05-25

**Authors:** Patrapan Juntavee, Hattanas Kumchai, Niwut Juntavee, Dan Nathanson

**Affiliations:** ^1^Department of Restorative Sciences & Biomaterials, Boston University Goldman School of Dental Medicine, Boston, MA, USA; ^2^Department of Prosthodontics, Faculty of Dentistry, Khon Kaen University, Khon Kaen, Thailand

## Abstract

**Objective:**

This study evaluated the effect of ceramic surface treatments on bond strength of metal brackets to machinable ceramics and veneering porcelain using different adhesive resins. *Materials and methods*. Machined ceramic specimens (10 × 10 × 2 mm) were prepared from Vitablocs mark II (Vita) and IPS e.max® CAD (Ivoclar). Layered porcelain fused to metal (IPS d.Sign®, Ivoclar) was used to fabricate PFM specimens (*n* = 60/group). Half of specimens were etched (9.6% HF, 15 sec), and the rest were nonetched. Three resin bonding systems were used for attaching metal brackets (Victory series™ APC II, 3M) to each group (*n* = 10): Transbond™ XT (3M), Light Bond™ (Reliance), or Blugloo™ (Ormco), all cured with LED curing unit (Bluephase G1600, Vivadent) for 50 s each. Specimens were immersed in deionized water at 37°C for 24 hours prior to shear bond testing (Instron) at crosshead speed of 0.5 mm/min. Debond surface of ceramic and bracket base was examined for failure mode (FM), Ceramic Damage Index (CDI), and Adhesive Remnant Index (ARI). ANOVA and post hoc multiple comparisons were used to analyze the differences in bond strength. The chi-squared test was used to determine significance effect of FM, CDI, and ARI.

**Results:**

Significant differences in shear bond strength among group were found (*p* ≤ 0.05) related to ceramic, surface treatment, and resin cement.

**Conclusion:**

Bond strength of bracket to ceramic is affected by type of ceramic, resin cement, and ceramic surface conditioning. Etching ceramic surface enhanced ceramic-bracket bond strength. However, bond strengths in nontreated ceramic surface groups were still higher than bond strength required for bonding in orthodontic treatment.

## 1. Introduction

Ceramic and metal-ceramic restorations are widely used as restorative materials for restoring damaged or missing teeth in form of veneers, crowns, and bridges because of their aesthetic appearance, outstanding mechanical properties, and biocompatibility [1]. The increasing demand for better dental restorations both aesthetic and function has caused the development of more advanced ceramic systems. These ceramics systems are marketed in several forms based upon methods in fabricating restoration such as conventional hand-condensed ceramic for fabrication metal ceramic restorations, heat-pressed ceramics, and machinable ceramic for CAD-CAM. The ceramic restorations are nowadays more often found in adult orthodontic patients who asking for orthodontic treatment. There is an increasing likelihood that orthodontic brackets and attachments to be placed to patients who already had ceramic restorations [2]. Many questions arise when bonding orthodontic attachments, like what types the existing ceramic restoration, which procedure should be applied for bonding brackets to ceramic restorations, and what will happen to the ceramic surface after debond.

Bond strength of brackets to ceramic restorations depends on many variables including type of ceramic, type of bracket, type of adhesive material, and surface conditioning method [3–5]. The composition and material property have a significant role on bond strength. Bonding of bracket to conventional feldspathic porcelain is a predictable and reliable procedure [6, 7].

The mechanical approach that alters ceramic surface in order to enhance bond strength can be achieved by roughening the ceramic surface, e.g., with diamond bur, with sandpaper discs, with laser irradiation, or by sandblasting with aluminum oxide particles [8]. However, these procedures introduced a destructive effect on the ceramic surface by reducing ceramic surface integrity [8, 9]. The chemical approaches to alter the ceramic surface can be achieved by either etching or changing ceramic surface for bonding affinity for adhesive resin materials [10] to adhere to ceramic restoration. Hydrofluoric acid (HF) and acidulated phosphate fluoride (APF) were reported to be able to facilitate micromechanical retention [11–13]. Nevertheless, all these procedures damage the glazed surface of restoration. The other chemical approach can be achieved by use of silane coupling agents. Silane coupling agent can form polysiloxane networks or hydroxyl groups covering the silica surface of ceramic [10, 14, 15]. This results in forming a bridge between ceramic surface and adhesive resin layer [16].

The purposes of this study were to compare the effects of ceramic surface conditioning to machinable ceramic restorative materials and conventional ceramic veneer metal and effect of different adhesive resins on shear bond strength between restorative materials and orthodontics brackets. The null hypothesis is that there is no effect from types of restorative materials and types of resin cement on shear bond strength between restorative materials and orthodontics brackets.

## 2. Materials and Methods

**Figure 1:**
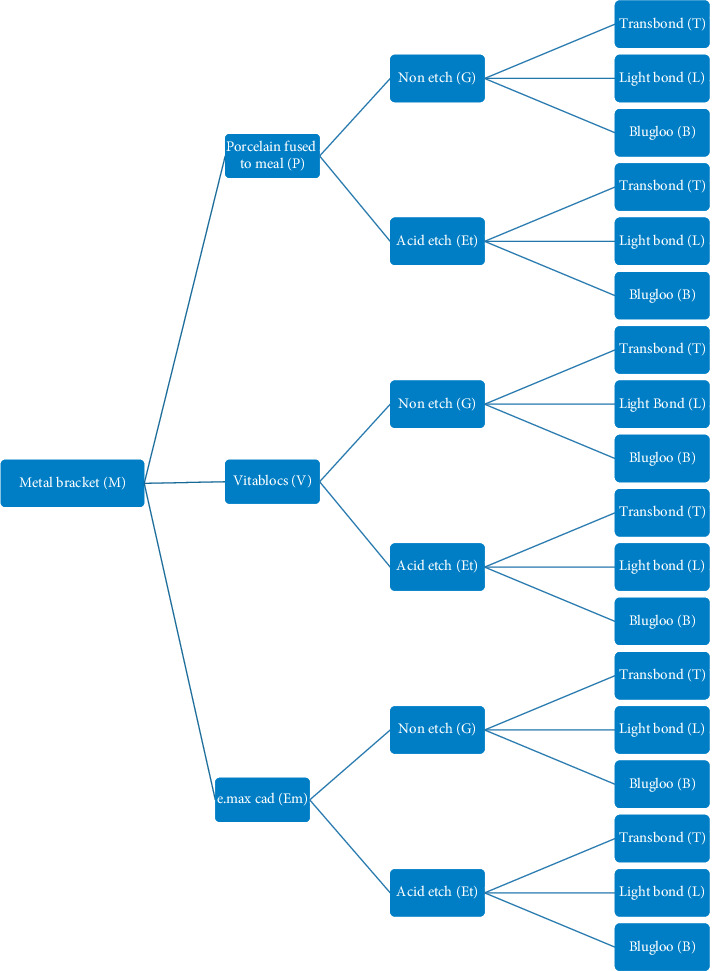
Sample tree design for metal bracket bonded on different surfaces treated of conventional and machinable ceramic.

The composition, trade name, and manufacture of ceramic and adhesive resin cements are presented in Table 1. The studies are designed as shown in Figure 1.

### 2.1. Preparation of Ceramic Specimens

Machinable ceramic specimens were prepared from VITABLOCS™ Mark II (Vita, Vident Co.) and IPS e.max® CAD™ (Ivoclar-Vivadent Inc.). The layered porcelain fused to metal specimens were prepared from IPS d.SIGN® porcelain veneered on D.SIGN 10® metal ceramic alloy (Ivoclar-Vivadent Inc.) (*n* = 60/each type of ceramic specimen).

#### 2.1.1. Preparation of Machinable Ceramic Specimens

The VITABLOCS™ Mark II and IPS e.max® CAD ceramic specimens were cut from the machinable ceramic blocks into square shape of (length × width × thickness) 10 × 10 × 2.2 mm by using a sectioning machine (Isomet 2000®, Buhler Co.). Then, the ceramic specimens were polished by using silicon carbide paper with 1200 roughness in the polishing machine (ECOMET 3®, Buhler Co.). The diamond suspension (Metadi®, Buhler Co.) with the polishing wheel was used to produce the smooth surface and to the final dimension of (length × width × thickness) 10 × 10 × 2.0 mm.

For VITABLOCS™ Mark II specimens, they were then glazed in the porcelain furnace (Programat CS®, Ivoclar-Vivadent) according to the manufacturer's firing cycle (Table 2). Final thickness of 2.0 mm was achieved for all specimens with glazed surface. The e.max® CAD specimens were crystalized and glazed in the porcelain furnace according to the manufacturer's firing cycle as shown in Table 2 to produce glazed surface to the ceramic specimen. Each surface should derive for final thickness of 2.0 mm for all specimens with glazed surface.

#### 2.1.2. Preparation of Ceramometal Specimens

The ceramometal specimens were prepared based on conventional porcelain fused to metal restoration technique. Base metal alloy specimen in square shape of tiles measuring 10 × 10 × 0.228 mm were casted to form base metal (IPS d.sign 10®, Ivoclar-Vivadent Inc.). The metal “substructures” were sandblasted with 50 microns aluminous oxide abrasive and cleaned in an ultrasonic cleaning machine in distilled water. The opaque porcelain (IPS d.SIGN®, Ivoclar-Vivadent Inc.) was applied to each metal surface using the brushing technique and then fired in a porcelain furnace according to firing temperature recommended by the manufacturer. The thickness of fired opaque porcelain must be 0.3 mm after firing no more than twice. The body porcelain shade A3 (IPS d.SIGN® (Ivoclar-Vivadent Inc.) was condensed onto the fired opaque porcelain surface using the porcelain condensing machine (Shofu Co.) and fired in the porcelain furnace according to firing temperature recommended from the manufacturer. The final dentine porcelain thickness of 1.5 mm. is produced upon firing dentine porcelain no more than twice. The “body porcelain” was polished and glazed according to the manufacturer's recommendation. The final conventional ceramometal specimens were constructed to have a dimension of (width × length × thickness) 10 × 10 × 2.0 mm.

### 2.2. Ceramic Surface Treatment Technique

The randomly assigned ceramic in each group was surface treated differently according to the technique tested.

#### 2.2.1. Nonetched Ceramic Surface (Glazed Surface)

The ceramic specimens in these groups were only cleaned with the distilled water and dried out with absorbing tissue paper.

#### 2.2.2. Etched Ceramic Surface for 15 Seconds

The ceramic surfaces were etched with 9.5% hydrofluoric acid gel (Ultradent® Etch, Ultradent product Inc.) for 15 seconds by continuously agitating with an applicator brush. The acid was then rinsed from the ceramic surface and dried with compressed air from the triplex syringe for 10 seconds.

### 2.3. Bonding of Orthodontic Bracket to Ceramic Surface

The ceramic specimens from each group (10 samples each) were bonded with metal bracket Victory™ series APC II (3M Unitek, Monrovia, CA, USA) by using one of the orthodontics adhesive resins that were Transbond™ XT (3M Unitek), Light Bond™ (Reliance Orthodontic Products, Inc.), and Blugloo™ (Ormco Co.). The bracket was bonded firmly into position on the ceramic specimen with bracket pliers by applying a force approximately about 5 N. The cement film thickness of adhesive resin was controlled to be 25 microns by using a digital veneer caliper. Excess adhesive was removed with an explorer without touching the bracket. Then, the adhesive resin was light cured for 50 seconds (10 seconds on each side and 10 second on the top of bracket) by using a LED visible light-curing unit (Bluephase® G-1600 (Ivoclar-Vivadent) with an intensity of 1100 mW/cm^2^. Sixty orthodontic brackets were bonded with each type of resin adhesive. Totally, 180 orthodontic metal brackets were bonded with adhesive resin. Then, all samples were stored in distilled water at 37°C for 24 hours prior to the test for the shear bond test.

### 2.4. Shear Bond Strength Testing

**Figure 2:**
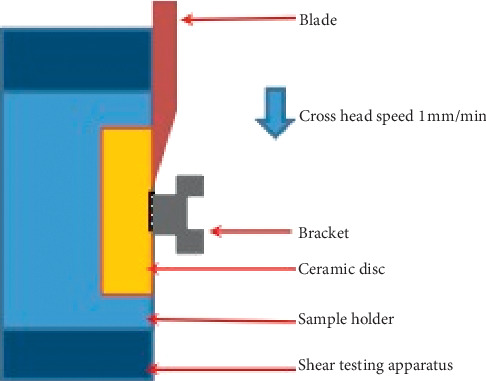
Load applied through adhesive/ceramic interface at 0.5 mm/min crosshead speed.

The sample was mounted in the sample holder, placed into the testing apparatus, and secured with a stabilizing screw in the testing jig that was mounted in an Instron 5566A Universal Testing machine (Instron, Norwood, Massachusetts, USA). A straight knife-edged chisel blade was applied the vertically loading force at a crosshead speed of 0.5 mm/minute (ISO TR 11405, 1994) directly applied to the bracket-ceramic interface. The load was applied until bond failure occurred as shown in Figure 2. The failure loads (*N*) were recorded and calculated for bond strength in mega Pascal (MPa).

### 2.5. Evaluation of Fracture Sites

Each debonded bracket base and ceramic surface were examined visually and under a light-optical stereomicroscope (Nikon Co.) at 10x magnification in order to evaluate the mode of failure and to assess the damage to the ceramic. The mode of failure was observed. The mode of bond failure (FM) was classified as one of the following failure types [17]:

1. *Type I*. Failure at bracket—adhesive resin interface: ninety per cent or greater of the bracket base was exposed and 10 per cent or less of the bonded ceramic was free of adhesive
2. *Type II*. Failure at adhesive resin—ceramic interface: ten per cent or less of the bracket base was exposed and 90 per cent or more of the bonded ceramic was free of adhesive
3. *Type III.* Failure of the bracket itself: fracture of the bracket during removal left part of the bracket still bonded to the ceramic.
4. *Type IV*. Failure of the ceramic itself: a portion of the ceramic was removed with the bracket base without loss of more than 10 percent of the adhesive from bracket base.
5. *Type V.* Combination failure (mixed): less than 90 per cent but more than 10 per cent of the bracket base was exposed or more than 10 per cent, but less than 90 per cent of the bonded ceramic surface was free of adhesive

The amount of ceramic surface alteration or damage was examined and classified using “Ceramic Damage Index (CDI)” proposed by the authors as follows:

1. No detectable ceramic surface damage. Ceramic surface intact or in the same condition as before bonding procedure.
2. No detectable ceramic surface damage. Ceramic surface intact or in the same condition as before bonding procedure.
3. Localized detectable ceramic surface alteration limited to superficial surface observed under microscope.
4. Generalized detectable ceramic surface alteration limited to superficial surface observed under the microscope.
5. Localized detectable ceramic surface damage observed by visual which features significant damage that require restoration of defect by resin composite.
6. Generalized detectable ceramic surface damage observed by visual which features significant damage that requiring restoration of defect by resin composite.
7. Localized ceramic surface damage or fracture.
8. Generalized ceramic surface damage or fracture.

In addition, the amount of adhesive remnant left on the ceramic surface was classified using modified “Adhesive Remnant Index (ARI)” and was scored as follows [18]:

1. No adhesive resin cement remained on the ceramic
2. Less than half of adhesive resin cement remained on the ceramic
3. More than half of adhesive resin cement remained on the ceramic and
4. All adhesive resin cement remained on the ceramic, along with a distinct impression of the bracket mesh

### 2.6. Statistical Analysis

Shear bond strength testing was analyzed using analysis of variance (ANOVA) both one-way and two-way to determine significant differences among various groups. The post hoc multiple comparison test (Bonferroni) was used to identify which of the groups was significant difference. The chi-squared test was used to determine significant differences in the mode of failure (FM), ceramic damage index (CDI), and adhesive remnant index (ARI) upon each factor. Significance differences for all statistical tests were determined at 95% level of confidence.

## 3. Results

**Figure 3:**
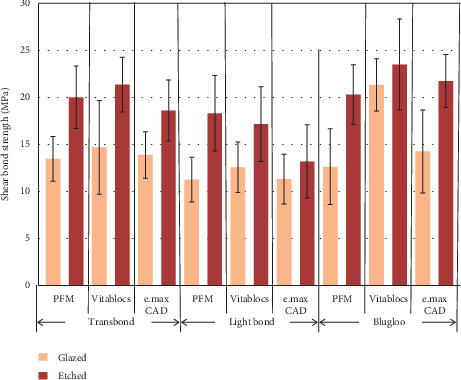
Histogram presenting shear bond strength of metal brackets to the ceramic materials tested with three cements and etching effects.

The results of the shear bond strength test are described in Figure 3. The results of ANOVA are shown in Table 3. There were statistically significant differences in the shear bond strengths as a result of different types of ceramic materials, methods of ceramic surface treatment, and types of resin adhesive for bracket bonding (*p* < 0.05).

**Figure 4:**
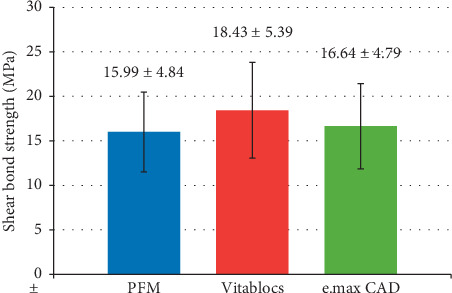
Shear bond strength of metal bracket bonded to the three different types of ceramic materials.

There were significant effects on shear bond strength of metal bracket to the ceramic veneering materials due to the factor of different types of ceramic materials, surface treatment, resin bonding materials, interaction between types of ceramic materials, and types of adhesive resin cement (*p* < 0.05). The mean shear bond strength of metal bracket bonded to VITABLOCS™ Mark II was higher than bonded to IPS e.max® CAD and bonded to IPS d.SIGN® porcelain as shown in Figure 4 (*p* < 0.05). Bonferroni post hoc multiple comparison indicated that the mean shear bond strength of metal bracket bonded to IPS d.SIGN® porcelain for PFM was significant lower than the mean shear bond strength of metal bracket bonded to VITABLOCS™ Mark II ceramic materials (*p* < 0.05). Also, the mean shear bond strength of metal bracket bonded to IPS e.max® CAD ceramic reveals significantly lower than the mean shear bond strength of metal bracket bonded to VITABLOCS™ Mark II ceramic materials (*p* < 0.05).

Bonferroni post hoc multiple comparisons indicated that the metal bracket bonded to ceramic materials using Blugloo™ cement revealed significantly higher shear bond strength than that of the metal bracket bonded to ceramic materials using Transbond™XT cement (*p* < 0.05). The metal bracket bonded to ceramic materials using Transbond™XT cement revealed significant higher shear bond strength than when using Light Bond™ cement (*p* < 0.05). The *t* test indicated significance effect on the shear bond strength as a result of ceramic surface treatment (*p* < 0.05).

The patterns of bond failure were mainly revealed in two types that were adhesive failure between metal bracket base and resin adhesive bonding interface and mixed mode of failure demonstrated as some of the resin remaining on both bracket and ceramic surface. The nature of bond failure was significantly different in the pattern of failure among groups tested (*p* < 0.05).

The amounts of ceramic surface alteration or damage were classified using the Ceramic Damage Index (CDI). The amount of ceramic surface alteration or damage indicated that there were no detectable surface alteration or damage to the surface of ceramic in all groups of specimens except for group 5 (PE_t_L) and group 9 (VGB) that had one of sample in both groups that exhibited slight ceramic surface alteration as localized area of ceramic surface damage. However, most of the sample in each tested group did not induce surface alteration or defect after debond. The statistics analyses were determined for the factors that influenced to ceramic surface alteration. Ceramic surface alteration revealed no significant difference among groups (*p* > 0.05). The statistics indicated that there was no statistically significant influence on ceramic surface damages due to type of ceramic for bonding bracket (*p* > 0.05).

**Figure 5:**
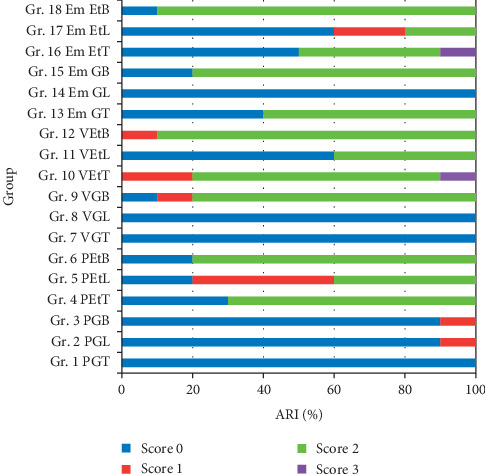
Frequency distribution (%) of type of Adhesive Remnant Index (ARI) score.

The amounts of adhesive remnant left on the ceramic surface were classified using the Adhesive Remnant Index (ARI). The frequency distribution of the amount of adhesive remnant was determined in percentage for each group as indicated in Figure 5. The patterns of adhesive remnant revealed a difference in the amount of remaining resin adhesive on the surface of ceramic according to the adhesive remnant index classification.

The statistics analysis was determined for the factors that influenced adhesive remaining on the surface of ceramic. Adhesive remaining on the surface of ceramic was significantly different among the groups (*p* < 0.05). The statistics also indicated that there was no statistically significant influence on the adhesive remaining on the surface of ceramic due to type of ceramic for bonding bracket (*p* > 0.05). There was statistically significant influence on the adhesive remaining on the surface of ceramic due to method of ceramic surface treatment prior to bonding (*p* < 0.05).

## 4. Discussion

The ideal orthodontic bonding should ensure that the bracket remain attached to the tooth surface for the duration of treatment, withstand application of forces to achieve tooth movement, and be easily removed at the end of treatment without damaging to the tooth surface. Once the bracket was bonded to ceramic restoration, the extremely high bond strength between bracket to ceramic restorative material may not usually required, but the optimal bond strength that provide sufficiently strong to endure orthodontic and masticatory force during the period of orthodontic treatment needs to be achieved yet being sufficiently weak to permit removal of the bracket from ceramic restoration without damaging the ceramic surface. There are few scientifical recommendations in the literature on what the minimum orthodontic bracket shear bond strength should be. There was a study reporting that the maximum orthodontic force of 14 kg/cm^2^ should be applied to the orthodontic appliance [19]. However, there are a number of studies frequently suggesting that the clinically adequate bond strength for a metal orthodontic bracket bonded to enamel should be between 6–8 MPa. Thus, the bond strength of 6–8 MPa was used as standard bond strength for most researchers and clinicians to refer to their studies and in clinical practice [20].

The result demonstrated that acid etching with 9.5% hydrofluoric acid on ceramic restorative materials enhanced the bond strength for both ceramic bracket and metal bracket bond to ceramic materials for each type of adhesive resin bonding materials. In the ceramic group that was etched with hydrofluoric acid, a number of specimens had adhesive failures between of resin adhesive and ceramic surface, some specimens had cohesive failure, and the bond strength was higher than that of the nonetched groups. The possible contribution of the ceramic primer and the bonding agent may have had an impact on the results. This may also be due to the differences in the luting agent used. The least favorable bond strength was obtained with 11.26 MPa in the group of metal bracket bonded to nonetched IPS d.SIGN® porcelain veneering metal using Light Bond™ (PGL group); however, the type of bond failures in this group was the desirable mode.

The result of this study introduced a method for bracket bonding to ceramic restorative materials based on nonetched ceramic surface using adhesive resin with silane to achieve appropriate bond strength. However, to be assured of the bonding strength of the bracket to ceramic restoration, minimal etched ceramic surface for 15 seconds is still recommended. Hydrofluoric acid etching of ceramic surface for 15 seconds was recommended from this study in order to maintain the integrity of ceramic surface to reduce the surface alteration. Care has to be made when utilizing hydrofluoric acid etching as it is known to be harmful to soft tissues [21].

In this study, ARI scores indicated that there was higher frequency of bond failure at the ceramic-adhesive resin interface the groups of ceramic that were nonetched surfaces. However, in the group where ceramic surfaces were etched, there were high frequency of mixed mode failure occurred. The scanning electron micrographs of failure surface of specimens in each group also confirmed this finding. This type of failure in the mixed mode shows that the chemical and mechanical bonding was equal to or exceeded the cohesive strength of the adhesive resin.

As long as there was no damage on the ceramic surface or the damage can be repaired easily and the remaining adhesive resin remnant can be easily cleaned and finished as well as the bond strength achieved from this study is higher than that clinically needed, the regimen in this study is suitable to be applied for clinical practice. The results of this in vitro study may not fully apply to the clinical situation but can be used as guidance for the clinician to make their own decision for clinical practice. Further studies should be performed using porcelain previously exposed to the oral environment. In addition, this investigation only evaluated the shear forces during orthodontic treatment; hopefully, the result of the shear bond test could be generalized for torqueing forces and tensile forces. Thus, further experiment should also be investigated, especially the clinical study.

## 5. Conclusion

On the basis of the results of this study, the following conclusions were made:

1. The shear bond strength of orthodontic brackets bonded to three different ceramic surfaces using three different resin adhesives with either etched or nonetched ceramic surfaces was stronger than the shear bond strength suggested for orthodontic bonding in clinical practice.
2. Etched surface treatments provided significantly higher bond strength of bracket to ceramic materials than the nonetched ceramic surface treatment.
3. The mode of failure of the metal brackets bonded to ceramic predominately occurred at the ceramic-adhesive resin interface in case of ceramic surfaces not etched on the surface. However, there was increase in the mixed mode of failure which exhibited increasing cohesive failure in the resin adhesive in the groups that were etched on the surface.

## Figures and Tables

**Table 1:** List of dental ceramic and resin adhesive: type, composition, and manufacturers.

Materials	Type	Composition	Manufacturer
VITABLOCS™ mark II	Machinable ceramic	Feldspathic based ceramic	Vita, Vident Co., Brea, CA, USA
IPS e.max® CAD	Machinable ceramic	Lithium disilicate glass-ceramic	Ivoclar-Vivadent Inc., Amherst, NY, USA
IPS d.SIGN® porcelain	Conventional metal-ceramic	Fluoroapatite-leucite glass-ceramic	Ivoclar-Vivadent Inc., Amherst, NY, USA
Transbond™XT	Conventional hybrid	Bis-GMA, Bis-EMA, TEGDMA 73–77% silanated quartz and silica	3M Unitek, St. Paul, MN USA
Light Bond™	Conventional hybrid	UDMA, TEGDMA, sodium fluoride, 85% fused silica	Reliance, Itasca, IL, USA
Blugloo™	Conventional hybrid	Uncured methacrylate monomer, Inert material fillers, fused silica,	Ormco Corp., Glendora, CA, USA

Bis-GMA: bisphenol A glycidyl methacrylate; Bis-EMA: biphenyl A glycol dimethacrylate; TEGDMA: triethylene glycol dimethacrylate; UDMA: urethane dimethacrylate.

**Table 2:** Program for crystallization and glazing.

Program	*T*s	*S*	*R*_1_	*T*_1_	*H*_1_	*R*_2_	*T*_2_	*H*_2_	*V*_1_	*V*_2_	*L*	*L*_t_
Glazing VITABLOCS™ mark II	600	4	70	950	1	—	—	—	—	—	—	—

Crystallization and glazing IPS e.max® CAD	403	6	90	820	10	30	840	7	550–820	820–840	700	—

Metal oxidization IPS d.sign 10®	403	4	80	950	1	—	—	—	450–950	—	—	—

Firing and glazing IPS d.SIGN®,												
Opaque	403	6	80	890	1				450–889			
Dentin	403	6	60	870	1				450–869			
Glazing	403	4	60	870	1				450–869			

*T*_s_: starting temperature (°C), *R*_1_: rate of firing stage 1 (°C/min), *R*_2_: rate of firing stage 2 (°C/min); (°C/min), *T*_1_: final temperature stage 1 (°C), *T*_2_: final temperature stage 2 (°C), *S*: prefiring, *H*_1_: holding time stage 1 (min), *H*_2_: holding time stage 2 (min), *L*_t_: long term cooling, *V*_1_: vacuum starting temperature stage 1 (°C), *V*_2_: vacuum temperature stage 2, (°C).

**Table 3:** The statistic results of an analysis of variance (ANOVA) of shear bond strength upon each variable tested.

Source	SS	df	MS	*F*	*p* value
CERAMIC	296.796	2	148.398	12.210	0.000
SURFACE	1324.515	1	1324.515	108.977	0.000
CEMENT	758.385	2	379.192	31.199	0.000
CERAMIC^*∗*^SURFACE	62.675	2	31.337	2.578	0.079
CERAMIC^*∗*^CEMENT	208.197	4	52.049	4.282	0.003
SURFACE^*∗*^CEMENT	19.510	2	9.755	0.803	0.045
CERAMIC^*∗*^SURFACE^*∗*^CEMENT	113.792	4	28.448	2.341	0.057
Error	1968.953	162	12.154		

SS: sum of square; df: degree of freedom; MS: mean square; *F: F* value; *p* value: probability value.

## Data Availability

The data used to support the findings of this study are available from the corresponding author upon request.
